# The Species-Selective Activity of Peptiskin Is Associated with Cell Wall Affinity and Extracellular DNA Binding in *Staphylococcus aureus* and *Staphylococcus epidermidis*

**DOI:** 10.3390/microorganisms14102287

**Published:** 2026-10-08

**Authors:** Akihiro Michihara, Masato Tange, Hiroshi Matsuoka, Fangfang Wang, Xianting Lin, Jianzhong Yang

**Affiliations:** 1 Faculty of Pharmacy and Pharmaceutical Sciences, Fukuyama University, Fukuyama, Hiroshima 729-0292, Japanmatsuoka@fukuyama-u.ac.jp (H.M.); 2 Beauty Hi-tech Innovation Co., Ltd., Kobe International Business Center 605, Chuo-ku, Kobe, Hyogo 650-0047, Japan; wang.f@bhinova.com (F.W.); lin.t@bhinova.com (X.L.); yang.jz@bhinova.com (J.Y.)

**Keywords:** Peptiskin, *Staphylococcus aureus*, *Staphylococcus epidermidis*, extracellular DNA, skin microbiota regulation

## Abstract

Skin health depends on the balance of the commensal microbiota. Excessive proliferation of *Staphylococcus aureus* contributes to inflammatory skin diseases, such as atopic dermatitis, whereas *Staphylococcus epidermidis* helps maintain skin homeostasis. We previously reported that Peptiskin, a mixture of short-chain cationic oligopeptides containing arginine and lysine, selectively suppresses *S. aureus* while promoting *S. epidermidis* growth, although the underlying mechanism remains unclear. We investigated this mechanism by evaluating Peptiskin’s effects on pH, nutrient utilization, extracellular DNA (eDNA), membrane damage, cell wall binding, antibacterial activity, bacteriostatic activity, and intracellular gene expression. The species-specific effects were not fully explained by changes in pH or nutrient availability. In *S. aureus*, Peptiskin induced substantial membrane damage, as indicated by ~66% lactate dehydrogenase release, stronger cell wall binding, higher antibacterial activity, and reduced ATP synthase-related gene expression, consistent with bactericidal and bacteriostatic effects. Conversely, membrane damage was minimal in *S. epidermidis*, whereas Peptiskin showed distinct interactions with eDNA, and extracellular interactions may contribute to the distinct growth response. The species-specific activity of Peptiskin was associated with differences in cell wall affinity, eDNA interactions, membrane susceptibility, and intrinsic physiological responses, providing a potential strategy for selectively modulating the skin microbiota while suppressing *S. aureus*-dominant conditions.

## 1. Introduction

Atopic dermatitis is a chronic inflammatory skin disease characterized by severe itching and impaired skin barrier function. *Staphylococcus aureus* is frequently detected on the skin of affected patients [1,2]. In contrast, *Staphylococcus epidermidis*, a commensal skin bacterium, contributes to maintaining skin barrier function and eliminating pathogenic bacteria [3]. Studies have shown that *S. aureus* proliferation exacerbates skin inflammation, whereas *S. epidermidis* functions as an antibacterial factor [4]. Therefore, the relative balance between these two bacteria within the skin microbiota is thought to significantly influence skin pathologies. Conventional antibiotics non-selectively kill not only pathogenic bacteria but also commensal bacteria, disrupting the balance of the skin microbiota and potentially increasing the risk of antibiotic resistance and relapse [5,6]. Therefore, selective strategies that suppress pathogenic bacteria while preserving or actively utilizing commensal bacterial functions are needed. *S. aureus* and *S. epidermidis* belong to the same genus and are closely related species that exhibit contrasting characteristics of pathogenicity and symbiosis, making them suitable models for investigating species-specific response mechanisms.

In recent years, antimicrobial peptides have attracted attention not only as bactericidal factors but also as regulatory factors involved in maintaining skin microbiome homeostasis [7,8,9]. In this regard, bacterial biofilms and extracellular DNA (eDNA) have emerged as important factors that influence the localization and activity of antimicrobial peptides [10,11]. Bacterial eDNA contributes to structural stabilization and antimicrobial resistance as a matrix component of biofilms [10,11]. Furthermore, eDNA co-localizes with actin and contributes to the formation and maintenance of biofilm structure [12]. In addition, eDNA can be degraded by bacteria and utilized as a nutrient source [13]. These findings indicate that eDNA plays a multifaceted role within biofilms, functioning as a structural scaffold (a base that spatially positions and anchors molecules while regulating their interactions and functional expression) and as a nutrient source through its degradation. Because eDNA is a polymer with a strong negative charge derived from its phosphate groups, it is thought to selectively accumulate and retain surrounding cations. Therefore, the localization, effective concentration, and antimicrobial activity of cationic antimicrobial peptides may be spatially regulated through electrostatic interactions with eDNA.

Cationic antimicrobial peptides, which are rich in basic amino acids such as arginine and lysine, are known to induce membrane damage through electrostatic interactions with bacterial cell membranes and exhibit broad-spectrum antimicrobial activity [14]. Although these peptides exhibit antimicrobial activity, even as relatively short-chain oligopeptides, many lack species specificity and have been reported to exert similar inhibitory effects against multiple Gram-positive microorganisms, including *S. aureus* and *S. epidermidis* [15]. While the antimicrobial potency of existing arginine/lysine-rich antimicrobial peptides has been widely investigated, very few studies have reported the induction of opposing biological responses in closely related bacterial species. In contrast, Peptiskin is a low-molecular-weight oligopeptide preparation composed of a mixture of short-chain cationic oligopeptides containing arginine and lysine, and shares similarities with conventional antimicrobial peptides in terms of amino acid composition and positive charge [16]. Unlike conventional antimicrobial peptides with a single defined amino acid sequence, Peptiskin consists of multiple short-chain oligopeptides with different sequences and molecular weights. This structural heterogeneity may allow Peptiskin to interact with bacterial surfaces and extracellular components, including eDNA, through multiple interactions. We previously reported that Peptiskin exhibits species-specific effects that are difficult to explain within the conventional framework of antimicrobial peptides, such as inhibiting the growth of *S. aureus* while promoting the growth of *S. epidermidis* [16,17]. Thus, Peptiskin may function not as an antimicrobial molecule that uniformly inhibits both bacteria but rather as a regulatory factor that elicits different biological responses depending on the bacterial species. However, the molecular mechanisms underlying these differential effects remain unknown.

The mechanisms of action of antimicrobial peptides have been studied in relation to changes in culture medium pH [18,19], interactions with eDNA [11,20], damage to cell membranes and cell walls [21], bacteriostatic effects [22], and differences in transcriptional responses [21,23,24]. It has also been suggested that eDNA released during cell lysis may serve as a nutrient source for microorganisms [13]. These findings indicate that the biological effects of antimicrobial peptides are multifaceted and cannot be explained by a single mechanism. Likewise, the species-specific action of Peptiskin is unlikely to depend on a single antimicrobial mechanism but is instead likely determined by the combined effects of extracellular environmental factors (pH and nutritional conditions), interactions with extracellular molecules (eDNA), and affinity for cell surface structures (cell wall and cell membrane). Importantly, the relative contributions of these factors may differ among bacterial species because of intrinsic differences in cell surface properties, cell envelope structure, membrane susceptibility, and eDNA-associated characteristics of the bacteria. For example, differences in surface charge and cell envelope properties may influence the interaction between Peptiskin and the bacterial surface, thereby affecting the extent of membrane damage. Similarly, differences in the abundance or characteristics of eDNA may alter the interaction and localization of Peptiskin in the extracellular environment, potentially contributing to differences in subsequent growth responses. Thus, the same Peptiskin exposure may result in different biological outcomes depending on the physiological characteristics of each bacterial species. Therefore, to understand the species-specific mechanism of action of Peptiskin, it is necessary to exclude the nonspecific effects of environmental factors and systematically evaluate its interaction with eDNA, its binding to the cell surface, membrane damage, and intracellular responses in an integrated manner.

This study aimed to elucidate the molecular mechanisms by which Peptiskin exerts opposing effects on *S. aureus* and *S. epidermidis*. By focusing on multiple levels, including environmental factors, extracellular molecules, cell surface structures, and intracellular responses, we conducted a multifaceted analysis of the differential responses of the two species. These factors may contribute differently to each species, and their combined effects may ultimately determine the species-specific growth response to Peptiskin.

## 2. Materials and Methods

### 2.1. Reagents

Peptiskin was purchased from Nikko Chemicals Co., Ltd. (Tokyo, Japan). The final concentration of Peptiskin used in this experiment was 0.08%. This concentration was prepared using a 40% Peptiskin formulation. Unless otherwise specified, commercially available, reagent-grade chemicals were used. 

### 2.2. Test Strains

*S. aureus* (NBRC 12732) and *S. epidermidis* (NBRC 12993) were used as test strains. Both strains were obtained from the National Institute of Technology and Evaluation Biotechnology Research Center (NBRC).

### 2.3. Culture Conditions

*S. aureus* and *S. epidermidis* were cultured under aerobic conditions at 37 °C using NBRC802 medium (1% hypopolypeptone, 0.2% yeast extract, 0.1% magnesium sulfate heptahydrate). The effects of pH, nutrient sources, and DNase I treatment on bacterial growth, as well as the bacteriostatic activity of Peptiskin, were evaluated using a modified broth microdilution method in 96-well plates, as described previously [25]. eDNA was extracted and quantified from the culture supernatants, and DNA–Peptiskin interactions were evaluated as described in Section 2.6 of this study. Membrane damage was assessed by measuring lactate dehydrogenase (LDH) leakage, as described in Section 2.8. Nutrient sources were tested in a concentration-dependent manner, whereas the other conditions were evaluated at fixed concentrations. A total of 180 µL of bacterial suspension and 20 µL of reagent or sterile water were added to each well of a 96-well plate to obtain a final volume of 200 µL. Cultures were grown using a Plate Shaker 00-NPS-2T (NICHIRYO Co., Ltd., Koshigaya, Saitama, Japan) at 37 °C. The turbidity of the culture medium was measured over time from 0 to 24 h using an Infinite 200 PRO (Tecan Trading AG, Männedorf, Switzerland) at an absorbance of 600 nm (OD_600_). The mean and standard deviation were calculated to evaluate bacterial growth.

### 2.4. Effect of pH

To explore whether changes in culture pH could account for the species-specific growth responses to Peptiskin, each strain was inoculated into NBRC802 medium and cultured at 37 °C. To reproduce the pH of *S. aureus*-containing culture medium at the time of Peptiskin addition (approximately 6.3), 50 mM Tris–HCl buffer (pH 6.0) was added in the absence of Peptiskin to adjust the overall pH of the medium to approximately 6.3. The initial pH of the culture medium was measured using a pH meter (HORIBA Navi pH METER F-52; HORIBA, Ltd., Kyoto, Japan), and the culture was maintained under the same conditions. The effect of pH on bacterial growth was evaluated by measuring the turbidity (600 nm) over time. This experiment was designed to assess the contribution of pH change alone to species-specific growth responses and was not intended to reproduce the entire physicochemical environment generated by Peptiskin treatment.

### 2.5. Evaluation of the Potential of Peptiskin as a Nutrient Source

To investigate whether *S. aureus* and *S. epidermidis* could utilize Peptiskin as a nutrient source, growth assays were conducted under low-nutrient conditions. NBRC802 medium was serially diluted with sterile water to final concentrations of 100%, 50%, 20%, 10%, and 0% (control). Bacterial cells were inoculated into each medium with or without Peptiskin, and the final concentration of Peptiskin was adjusted to 0.08% (*w*/*v*). The medium was incubated at 37 °C for 24 h. After incubation, turbidity (600 nm) was measured to compare bacterial growth under each condition. In addition, M9 minimal medium, lacking carbon and nitrogen sources, and Yeast Nitrogen Base, Dehydrated (Difco Laboratories, Detroit, MI, USA) for carbon assimilation test medium, which lacks amino acids (asparagine), were used. Bacterial cells were inoculated into each medium with or without Peptiskin and incubated at 37 °C. Turbidity (600 nm) was measured at the start of incubation and after 24 h to evaluate the effect of Peptiskin addition on bacterial growth. 

### 2.6. Extraction and Quantification of eDNA

To evaluate changes in eDNA levels following the addition of 0.08% Peptiskin, *S. aureus* or *S. epidermidis* were cultured in NBRC802 medium at 37 °C for 24 h in the presence or absence of 0.08% Peptiskin. After collecting the culture medium, bacterial cells were removed by centrifugation and DNA was extracted from the supernatant using the phenol/chloroform method. After ethanol precipitation of the DNA, it was dissolved in 10 µL of sterile water. The amount of recovered nucleic acid was estimated by measuring the absorbance at 260 nm using a NanoDrop One spectrophotometer (Thermo Fisher Scientific, Wilmington, DE, USA). To assess eDNA integrity, the extracted DNA was subjected to electrophoresis at 100 V for 30 min on a 3% agarose gel (tris–borate–EDTA [TBE] buffer), and the band pattern was visualized using a UV transilluminator (TPB-20LMP; ATTO Corporation, Tokyo, Japan). To investigate whether Peptiskin directly interacts with eDNA and affects its detection by agarose gel electrophoresis, we modified a previously reported method [26]. DNA extracted from the culture supernatant of *S. epidermidis* grown in the absence of Peptiskin was used for the assay. Four microliters of the DNA extract was mixed with 0, 0.5, 1, or 5 µL of 0.08% Peptiskin solution, and sterile water was added to adjust the final volume of each reaction mixture to 8 µL. The mixtures were incubated at 37 °C for 1 h and then subjected to electrophoresis on a 3% agarose gel under the conditions described above. Changes in the DNA band pattern following the addition of Peptiskin were evaluated to assess the interaction between eDNA and Peptiskin.

### 2.7. Evaluation of eDNA Involvement by DNase I Treatment

To investigate whether bacterial eDNA is involved in the action of Peptiskin, DNase I treatment experiments were conducted. *S. aureus* and *S. epidermidis* were inoculated into NBRC802 medium and cultured under the following four conditions: (i) untreated control group, (ii) group treated with 0.1 U DNase I alone, (iii) group treated with 0.08% Peptiskin alone, and (iv) group treated with both DNase I and Peptiskin. After 24 h of shaking the culture at 37 °C under each condition, turbidity (600 nm) was measured and compared with that of the untreated control group to evaluate the effect of DNase I treatment on the action of Peptiskin. This experiment was designed to examine whether the degradation of eDNA alters the growth response to Peptiskin, thereby assessing the potential involvement of eDNA in its species-specific activity.

### 2.8. Membrane Damage by Peptiskin

To evaluate whether Peptiskin causes loss of bacterial membrane integrity, membrane damage was assessed by measuring lactate dehydrogenase (LDH) leakage from bacterial cells. *S. aureus* and *S. epidermidis* were cultured in NBRC802 medium at 37 °C for 24 h in the presence or absence of 0.08% Peptiskin. After incubation, the culture supernatant and cell fractions were collected, and cell lysates were prepared. LDH activity in the culture supernatant and cell lysates was measured using a commercially available LDH cytotoxicity detection kit (Roche Diagnostics GmbH, Mannheim, Germany). The percentage of LDH leaked into the culture supernatant was calculated by dividing the LDH activity in the culture supernatant by the sum of the LDH activities in the culture supernatant and cell lysates. The degree of bacterial membrane damage caused by Peptiskin was quantitatively evaluated by comparing the LDH leakage rates in the presence or absence of Peptiskin. LDH leakage was used as an indicator of loss of bacterial membrane integrity [27].

### 2.9. Binding of Peptiskin to Cell Walls

To evaluate the initial interaction of Peptiskin with the bacterial cell surface, the binding of Peptiskin to the bacterial cell wall was assessed. Next, 3 mL of NBRC802 medium inoculated with *S. aureus* or *S. epidermidis* was cultured at 37 °C for 24 h, and the cells were harvested. After suspending the cells in phosphate-buffered saline (PBS), the cell wall fraction was prepared using a modified version of a previously reported method [28]. Specifically, sodium dodecyl sulfate (SDS; final concentration 0.1%) was added, and the suspension was incubated at 37 °C for 30 min, followed by the addition of proteinase K (100 µg/mL) and incubation at 37 °C for 1 h. The insoluble fraction was harvested as the cell wall fraction and resuspended in PBS. The protein concentration of the obtained cell wall suspension was measured using a NanoDrop One spectrophotometer (Thermo Fisher Scientific, Wilmington, DE, USA) and adjusted to 1 mg/mL. A total of 100 µL of cell wall suspension was dispensed into each well of an enzyme-linked immunosorbent assay (ELISA) plate and incubated overnight at 4 °C to adsorb cell wall components. After adsorption, the mixture was blocked at 37 °C for 1 h using PBS containing 5% (*w*/*v*) skim milk. Next, 0.08% Peptiskin solution or sterile water (control) was added to each well, and the mixture was incubated at 37 °C for 4 h. Unbound Peptiskin was recovered by washing three times with PBS, and the absorbance of each washing solution was measured at 200 nm (the Peptiskin absorption maximum). The amount of Peptiskin bound to the cell wall was calculated by subtracting the absorbance of the unbound amount (total recovered amount) from the initial absorbance before the addition of Peptiskin. Negligible absorbance at 200 nm was detected in the control group. This assay was performed to evaluate the initial interaction of Peptiskin with bacterial cell surface components.

### 2.10. Bactericidal Effect

To evaluate the antibacterial activity of Peptiskin against *S. aureus* and *S. epidermidis*, bacterial growth was monitored in the presence of Peptiskin. The bactericidal effect on the keratin film was evaluated according to a previously reported method [29]. As a modification, *S. aureus* or *S. epidermidis* attached to the keratin film were treated with sterile water (control) or 0.08% Peptiskin solution (500 µL) and incubated at 37 °C for 8 h. Subsequent washing, recovery of the remaining bacteria, serial dilution, culture on mannitol agar, and measurement of colony counts were performed as previously described. The colony-forming units (CFU) of the 0.08% Peptiskin-treated group were expressed as a percentage of that of the control group to evaluate bactericidal effect. This assay evaluated the effect of Peptiskin on bacteria that had been pre-attached to the keratin film and was not designed to evaluate de novo biofilm formation.

### 2.11. Bacteriostatic Activity

To evaluate whether the growth inhibition induced by Peptiskin was reversible, Peptiskin-treated bacterial cells were washed and subsequently cultured in Peptiskin-free medium. The bacteriostatic activity was evaluated in the presence or absence of Peptiskin. NBRC802 medium (200 µL) containing *S. aureus* was dispensed into 96-well plates with or without 0.08% Peptiskin and incubated at 37 °C for 24 h. After incubation, the turbidity (600 nm) was measured to evaluate the inhibitory effect of Peptiskin on bacterial growth. The culture was then centrifuged to collect the cells, and the residual Peptiskin was removed by washing with PBS. The washed cells were resuspended in fresh NBRC802 medium and dispensed into 96-well plates. After an additional 24 h of shaking incubation at 37 °C, turbidity (600 nm) was measured to evaluate the regrowth ability of the cells after Peptiskin removal. This experiment was performed to distinguish growth inhibition during Peptiskin exposure from the ability of Peptiskin-exposed cells to resume growth after Peptiskin removal.

### 2.12. RNA Extraction and Quantitative Reverse Transcription Polymerase Chain Reaction (qRT-PCR)

To evaluate the physiological and metabolic responses of bacteria following Peptiskin exposure, changes in the expression of selected genes were analyzed using RT-qPCR. *S. aureus* and *S. epidermidis* were cultured at 37 °C for 24 h with shaking, in the presence or absence of Peptiskin. Because bacterial growth of *S. aureus* was strongly suppressed in the presence of Peptiskin, the culture volume was increased to 5 mL to obtain sufficient bacterial biomass for total RNA extraction. After collecting 5 mL of culture medium, the cells were centrifuged and washed with PBS. The resulting cell pellet was suspended in a lysozyme-containing solution (1 mM ethylenediaminetetraacetic acid [EDTA], 0.5% Triton X-100, and 20 mM Tris-HCl) and allowed to stand at 4 °C. Equal amounts of RNase-free glass beads were added to each sample, and physical disruption was performed by vortexing. Because of the low RNA yield, the amount of lysozyme-containing solution was reduced in *S. aureus* cultured in the presence of Peptiskin. Total RNA was extracted using ISOGEN I (NIPPON GENE Co., Ltd., Tokyo, Japan) and purified according to the manufacturer’s protocol. The obtained RNA (2 µg) was treated with recombinant DNase I (RNase-free; cat. no. 2270A; Takara Bio Inc., Kusatsu, Shiga, Japan) at 0.6 U to remove genomic DNA, dissolved in sterile water, and the RNA concentration was measured using a NanoDrop One spectrophotometer. RNA purity was additionally assessed using the A260/A280 ratio. The mean A260/A280 ratios (*n* = 4) were 1.70 ± 0.06 for *S. aureus*, 1.80 ± 0.04 for *S. epidermidis*, 1.71 ± 0.08 for 0.08% Peptiskin-treated *S. aureus*, and 1.85 ± 0.02 for 0.08% Peptiskin-treated *S. epidermidis*. Reverse transcription was performed using equal amounts of purified total RNA as a template, followed by qRT-PCR. The expression levels of the candidate reference genes, 16S ribosomal RNA (16S rRNA) and DNA gyrase subunit B (*gyrB*), and target genes were evaluated in the presence or absence of Peptiskin. ATP synthase subunit alpha (*atpA*) was selected as a representative gene related to cellular energy metabolism, whereas arginine deiminase (*arcA*) and lysine decarboxylase (*ldc*) were selected as representative genes related to arginine and lysine metabolism, respectively. These genes were selected based on the composition of Peptiskin, which primarily consists of short-chain cationic oligopeptides containing arginine and lysine, and on the differences in bacterial growth, membrane damage, and physiological responses observed following Peptiskin treatment.

The primer sequences were as follows: 16S rRNA for *S. aureus* (F: GGACGAGAAGCTTGCTTCTCTGAT/R: TCACTTTTGAACCATGCGGTTCAA), *gyrB* (F: GAACGTGATGAAGAAAACGTTAG/R: AATCTTGCTACTTAAACCATAAC), *atpA* (F: TTCCATAACGGCGTACTTGG/R: CGATCCATTACACCAGTAGC), and *arcA* (F: CGCAGGTGCTAAGAGAAGAG/R: TTCTTCCTTACGTACCCCTG), and 16S rRNA for *S. epidermidis* (F: AGACGAGGAGCTTGCTCCTCTGAC/R: TCACTATTGAACCATGCGGTTCAA), *gyrB* (F: GATGAAAGAGAAGAGGAAGTTAG/R: AATCTTACTTTGCGTACCATAGC), *atpA* (F: GGAGAGGAAATGATAGGAAG/R: TTACCAGTTTGACGGTCACC), and *ldc* (F: CGGTTATCCAAGCATTTTCG/R: TTAGGGTATGTACAGATGGC).

### 2.13. Statistical Analysis

All experiments were performed independently at least three times. The results are presented as mean ± standard deviation. Student’s *t*-tests were used for comparisons between two groups, with a significance level of 5%. All statistical analyses were performed using Microsoft Excel for Microsoft 365 (Microsoft Corporation, Redmond, WA, USA).

### 2.14. Use of Generative Artificial Intelligence

OpenAI ChatGPT (GPT-5.6 Sol, OpenAI, San Francisco, CA, USA) was used only to assist in the preparation of the graphical abstract. The generated output was reviewed and edited by the authors before inclusion in the manuscript and was not used for data analysis, interpretation, or generation of scientific results.

## 3. Results

### 3.1. Effect of pH

**Figure 1:**
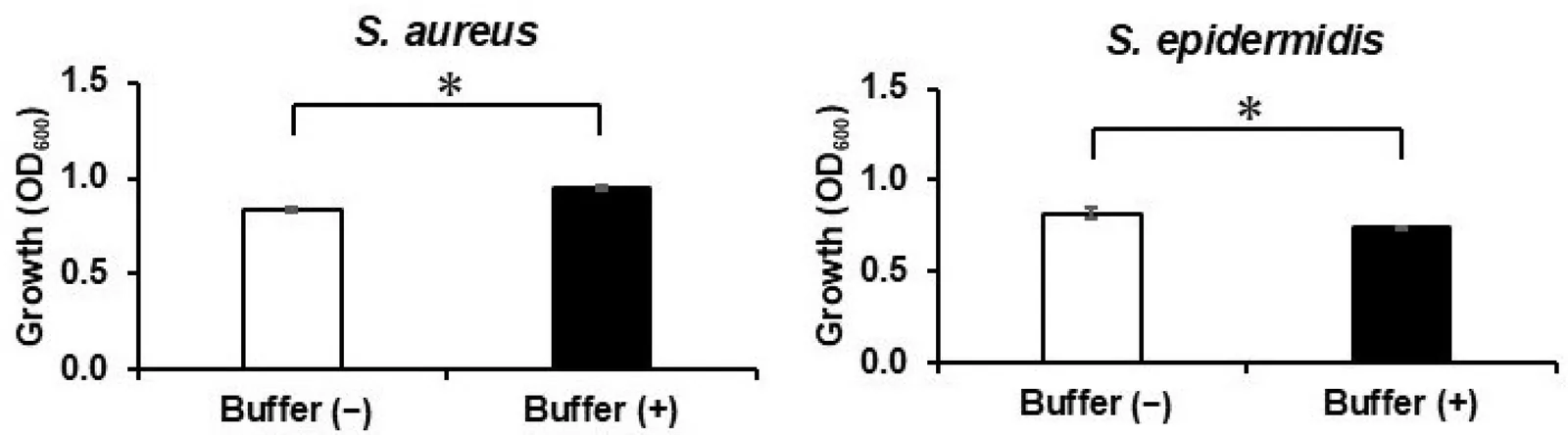
Effect of pH on the growth of *S. aureus* and *S. epidermidis*. The contribution of culture pH to species-specific growth responses was evaluated by comparing bacterial growth under unbuffered and pH-adjusted conditions in the absence of Peptiskin. To adjust the pH of the culture medium containing each bacterium to 6.3, 50 mM Tris–HCl buffer (pH 6.0) was added to the medium. The growth of *S. aureus* and *S. epidermidis* was evaluated by measuring turbidity. Cultures were incubated for 24 h under both unbuffered conditions (Buffer (−), □) and buffered conditions (Buffer (+), ■). Data are shown as mean ± SD (*n* = 4). * *p* < 0.05.

The pH of the culture medium containing each bacterium was measured in the presence of Peptiskin. The pH of the *S. aureus* and *S. epidermidis* culture media was 6.3 and 6.2, respectively. To investigate the effect of Peptiskin-induced changes in culture medium pH on bacterial growth, the pH of the culture medium was adjusted to 6.3 using 50 mM Tris-HCl buffer in the absence of Peptiskin, and both strains were cultured under these conditions. Under pH-adjusted conditions (pH 6.3), the growth of *S. aureus* increased significantly compared with that under unadjusted conditions, whereas the growth of *S. epidermidis* decreased significantly (Figure 1). Thus, adjusting the culture pH alone affected the growth of the two bacterial species in different directions. However, these responses did not fully reproduce the species-specific growth responses observed in the presence of Peptiskin, suggesting that changes in pH alone cannot fully account for the species-specific effects of Peptiskin on bacterial growth.

### 3.2. Effect of Peptiskin as a Nutrient Source

**Figure 2:**
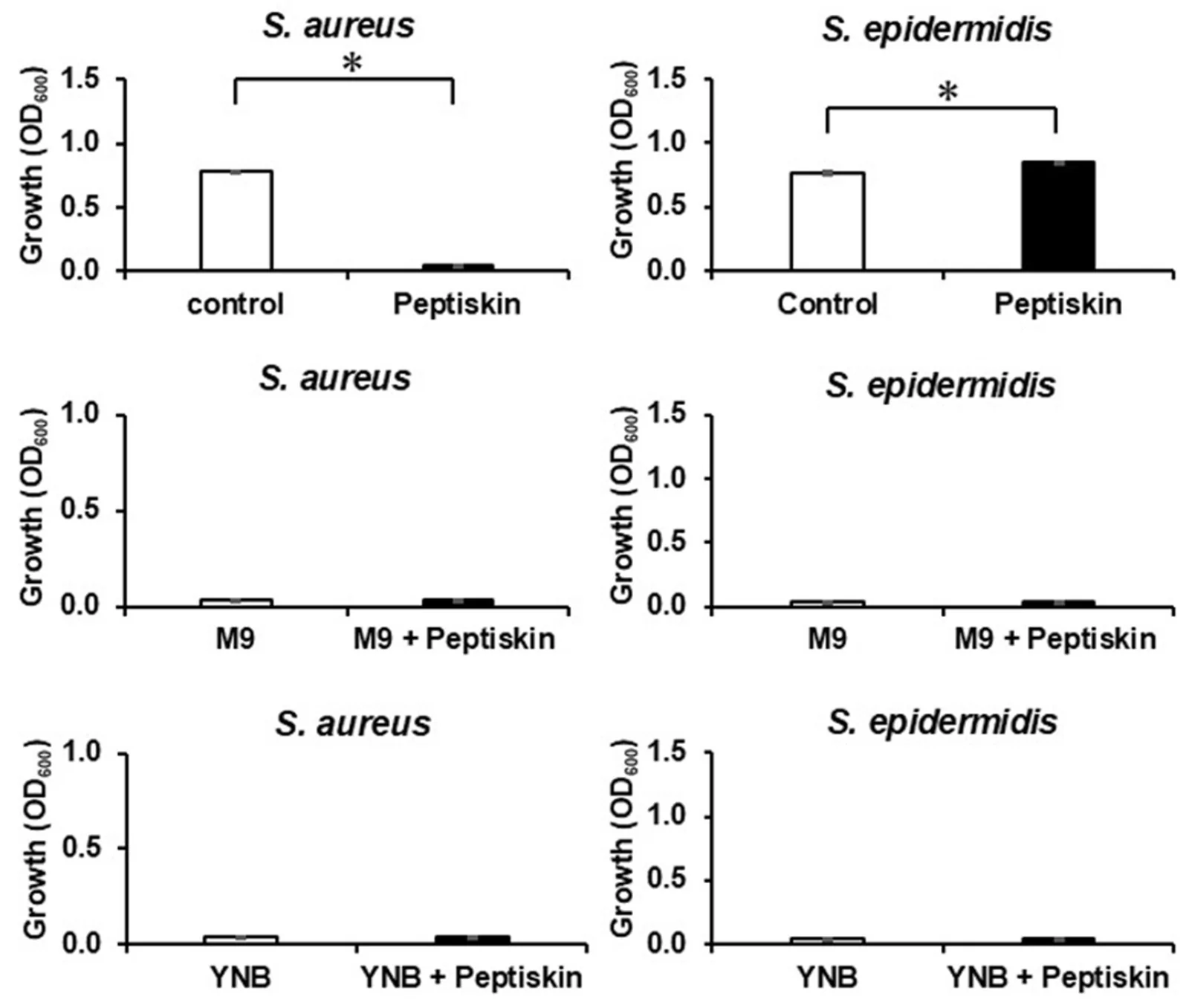
Evaluation of Peptiskin as a nutrient source for *S. aureus* and *S. epidermidis*. The growth of *S. aureus* and *S. epidermidis* was evaluated by turbidity in the absence and presence of Peptiskin (0.08%) using standard medium (control), M9 minimal medium (M9) without carbon and nitrogen sources, and Yeast Nitrogen Base for Carbon Assimilation Tests (Yeast Nitrogen Base [YNB]: Nitrogen) medium from which amino acids (asparagine) were removed. The cells were incubated for 24 h. White and black bars indicate the absence and presence of Peptiskin (0.08%), respectively. Data are shown as mean ± SD (*n* = 4). * *p* < 0.05.

Culturing was performed using medium concentrations of 50%, 20%, 10%, and 0% with the undiluted medium designated as 100%. In *S. aureus*, growth was significantly inhibited in the presence of Peptiskin at all medium concentrations (Table 1). In contrast, in *S. epidermidis*, significant growth promotion was observed in the presence of Peptiskin at all tested medium concentrations (100%, 50%, and 20%). No growth was observed for either species at concentrations below 10%. In the absence of Peptiskin, turbidity gradually decreased with decreasing medium concentrations for both species. Neither bacterium showed growth in M9 minimal medium lacking carbon and nitrogen sources or in Yeast Nitrogen Base medium lacking amino acids, regardless of the presence of Peptiskin (Figure 2).

### 3.3. Involvement of eDNA

**Figure 3:**
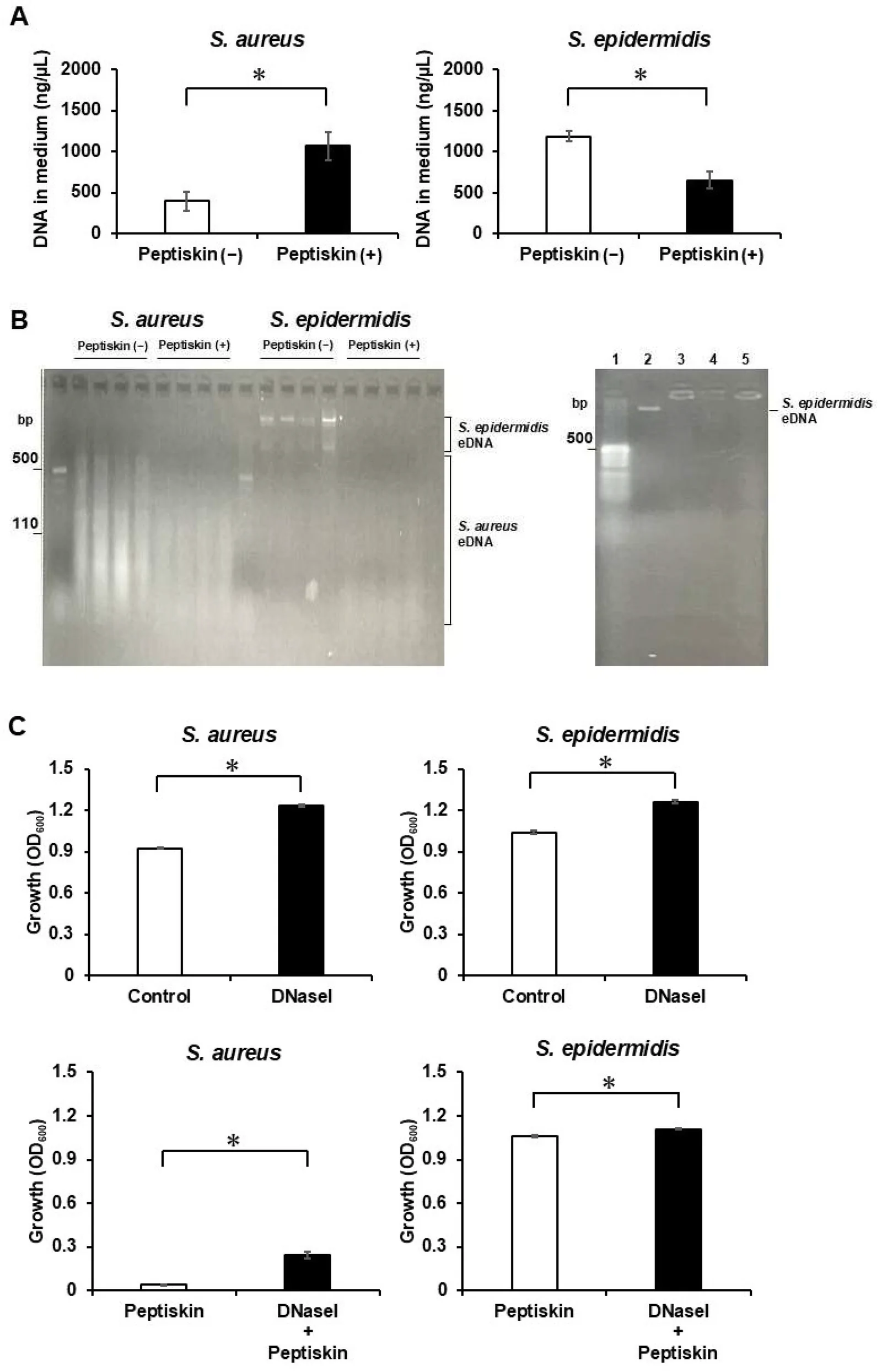
Interaction between extracellular DNA and Peptiskin and the effect of eDNA on bacterial growth. (**A**) After culturing in the absence (Peptiskin (−), □) and presence (Peptiskin (+), ■) of 0.08% Peptiskin, DNA was extracted from the culture supernatant, and the amount of eDNA released by each bacterium was evaluated by measuring the absorbance at 260 nm. (**B**) *S. aureus* or *S. epidermidis* was cultured for 24 h in the absence of 0.08% Peptiskin and in the presence of 0.08% Peptiskin. After culturing, the bacterial cells and supernatant were separated by centrifugation, and eDNA was recovered from the supernatant before being subjected to 3% agarose gel electrophoresis. In addition, DNA purified from the culture medium of *S. epidermidis* in the absence of Peptiskin was incubated with 0, 0.5, 1, or 5 µL of 0.08% Peptiskin at 37 °C for 1 h, followed by 3% agarose gel electrophoresis. Lane 1, DNA size marker; lane 2, DNA without Peptiskin; lanes 3–5, DNA incubated with 0.5, 1, or 5 µL of 0.08% Peptiskin, respectively. (**C**) Each strain was cultured under four conditions: untreated control (□), DNase I (0.1 U) alone (■), Peptiskin (0.08%) alone (□), and DNase I (0.1 U) plus Peptiskin (0.08%) (■). White and black bars indicate the absence and presence of DNase I (0.1 U), respectively. Turbidity (OD_600_) was measured after 24 h of incubation. Data are shown as mean ± SD (*n* = 4). * *p* < 0.05.

After 24 h of incubation with or without Peptiskin, eDNA was collected from the culture supernatant and quantified by measuring the absorbance at 260 nm. The amount of eDNA in *S. aureus* increased significantly in the presence of Peptiskin, whereas that in *S. epidermidis* decreased significantly by approximately 40% (Figure 3A). In the absence of Peptiskin, the amount of eDNA in *S. epidermidis* was higher than that in *S. aureus*. Agarose gel electrophoresis revealed ladder-like DNA fragments of 100–500 base pairs (bp) in *S. aureus* in the absence of Peptiskin; however, these bands were weaker in the presence of Peptiskin (Figure 3B). In contrast, distinct high-molecular-weight DNA bands of ≥10 kbp were observed in *S. epidermidis* in the absence of Peptiskin, but these bands disappeared in the presence of Peptiskin. To directly examine the interaction between Peptiskin and eDNA, eDNA purified from the culture medium of *S. epidermidis* in the absence of Peptiskin was incubated with increasing amounts of 0.08% Peptiskin (0, 0.5, 1, and 5 µL). High-molecular-weight DNA bands were detected in the absence of Peptiskin; however, the DNA bands disappeared upon the addition of 0.5 µL or more of 0.08% Peptiskin (Figure 3B). When the effects of DNase I treatment were examined in *S. aureus*, DNase I treatment alone significantly increased turbidity compared with the group without Peptiskin. Furthermore, when Peptiskin and DNase I were used together, turbidity significantly increased compared with the Peptiskin-treated group but did not reach the level of the untreated control group (Figure 3C). In *S. epidermidis*, DNase I treatment alone also significantly increased turbidity compared with that in the group without Peptiskin. Furthermore, when Peptiskin and DNase I were used together, a significant increase was observed compared with the Peptiskin-treated group; however, the increase was smaller than that observed in *S. aureus*.

### 3.4. Membrane Damage Due to LDH Release

**Figure 4:**
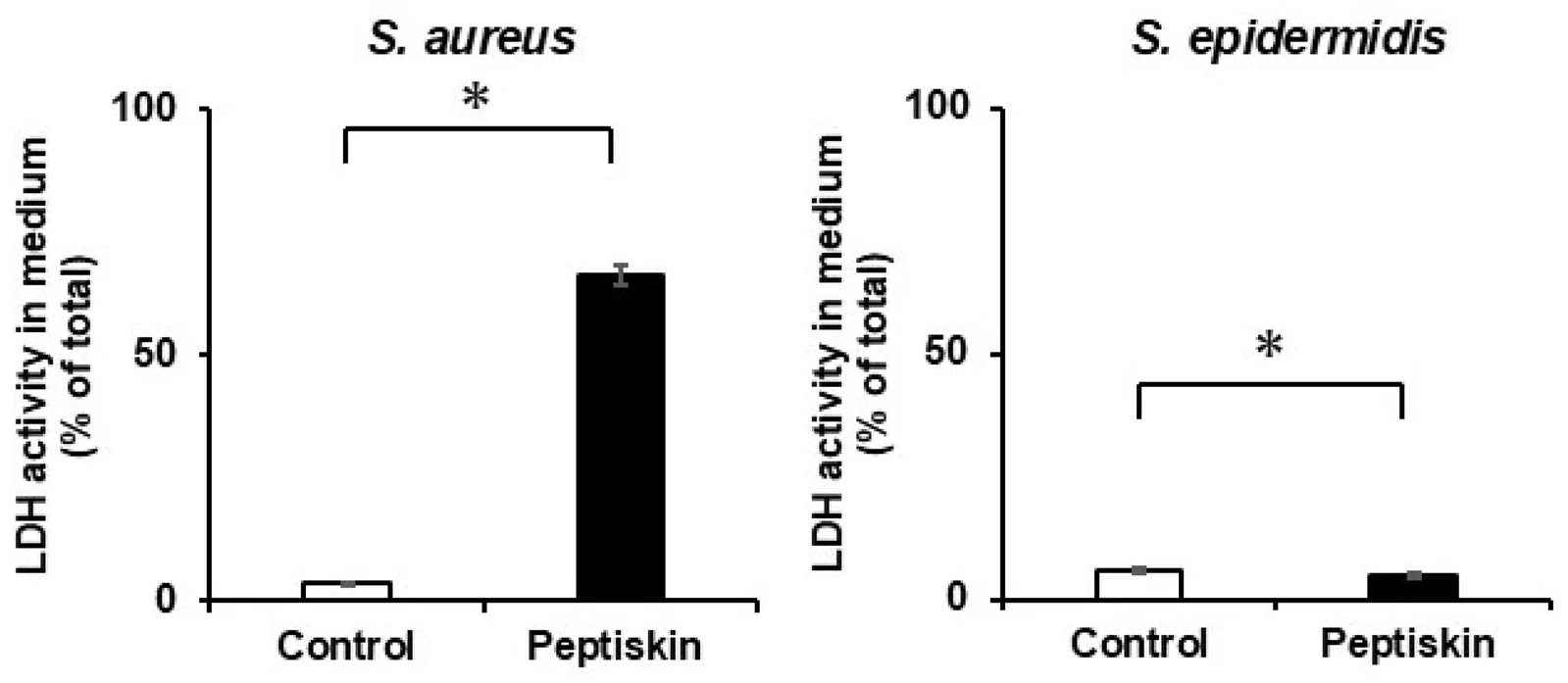
Evaluation of membrane damage in *S. aureus* and *S. epidermidis* after Peptiskin treatment. Membrane damage was evaluated by measuring lactate dehydrogenase (LDH) activity in the culture supernatant. *S. aureus* and *S. epidermidis* were incubated for 24 h in the absence (□) or presence (■) of Peptiskin (0.08%). After culturing, the bacterial cells and supernatants were separated by centrifugation, and the LDH activity in each fraction was measured. The LDH release rate was calculated by dividing the LDH activity in the supernatant by the sum of the LDH activities in the supernatant and the bacterial cell lysate. Data are shown as mean ± SD (*n* = 4). * *p* < 0.05.

When LDH activity in the culture supernatant was measured, *S. aureus* showed an LDH release rate of approximately 66% in the presence of Peptiskin, which was significantly higher than that in its absence (Figure 4). In contrast, *S. epidermidis* showed an LDH release rate of approximately 5%, regardless of the presence of Peptiskin.

### 3.5. Peptiskin Binding to Cell Walls

**Figure 5:**
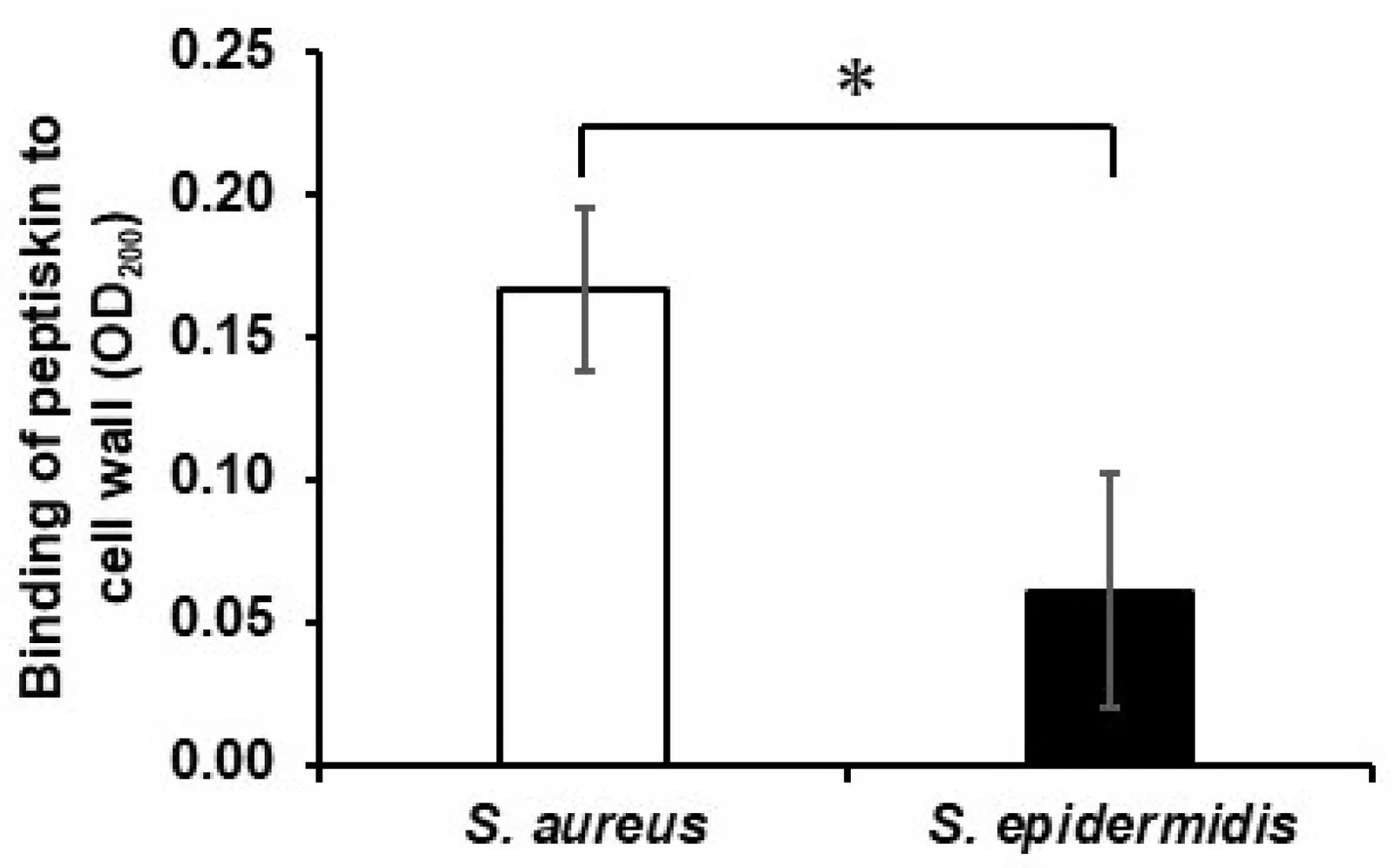
Binding of Peptiskin to cell wall fractions of *S. aureus* and *S. epidermidis*. Cell wall fractions from *S. aureus* (□) or *S. epidermidis* (■) were immobilized on an ELISA plate and incubated at 37 °C for 4 h in the presence of 0.08% Peptiskin or in its absence (sterile water, control). After washing, the amount of Peptiskin bound to the cell wall was evaluated by measuring the absorbance at 200 nm. Data are shown as mean ± SD (*n* = 4). * *p* < 0.05.

When the cell wall fraction was immobilized on an ELISA plate and its ability to bind Peptiskin was evaluated, the amount of Peptiskin bound to the cell wall of *S. epidermidis* was significantly lower than that bound to the cell wall of *S. aureus* (Figure 5).

### 3.6. Bactericidal Effect on Keratin Films

**Figure 6:**
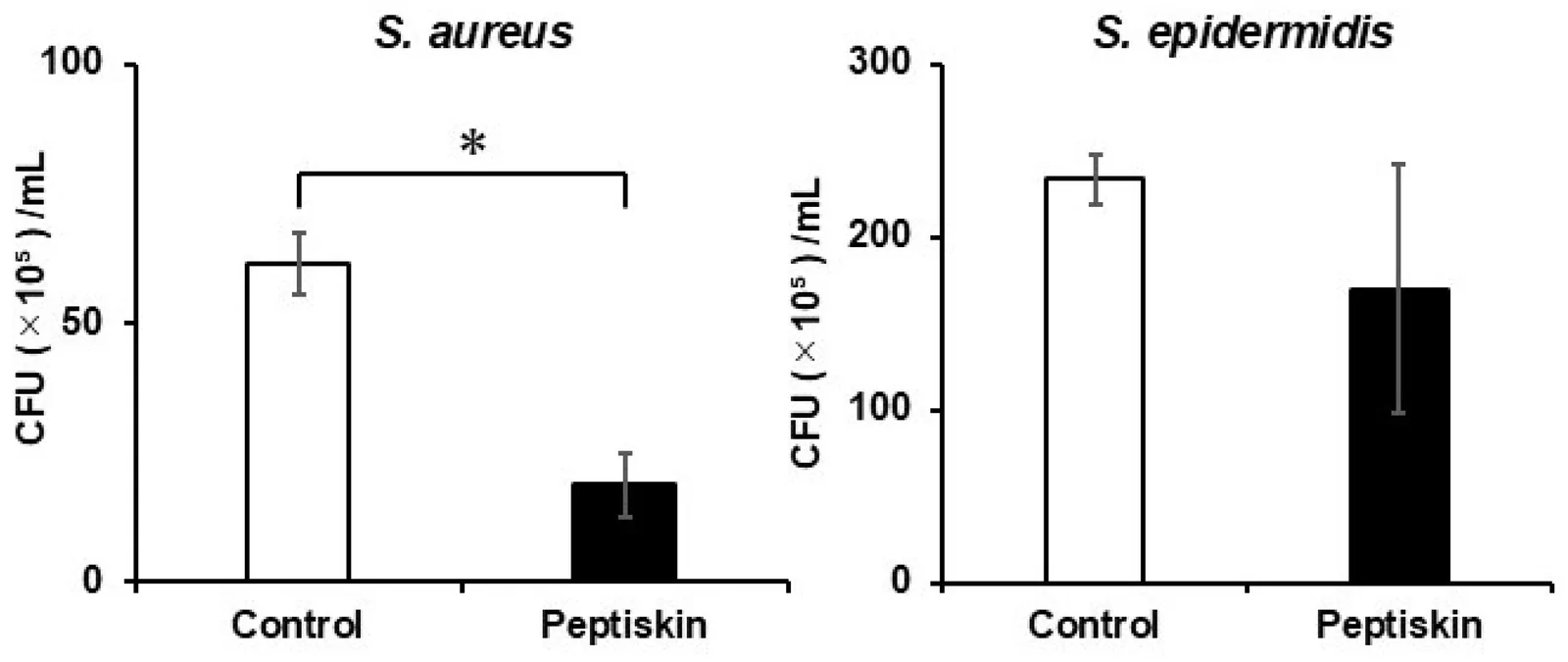
Bactericidal effect of Peptiskin on *S. aureus* and *S. epidermidis* attached to keratin films. *S. aureus* or *S. epidermidis* attached to keratin films were treated at 37 °C for 8 h with sterile water (control, □) or 0.08% Peptiskin (■). After treatment, the bacteria remaining on the film were collected, and the bactericidal effect was evaluated by determining colony-forming units (CFU). Data are shown as mean ± SD (*n* = 4). * *p* < 0.05.

When the bactericidal effect on bacteria attached to keratin films was investigated, the number of CFUs of *S. aureus* was significantly reduced in the presence of Peptiskin compared with that in its absence (Figure 6). In contrast, no significant change in the number of *S. epidermidis* CFUs was observed following Peptiskin treatment.

### 3.7. Bacteriostatic Effect of Peptiskin on S. aureus

**Figure 7:**
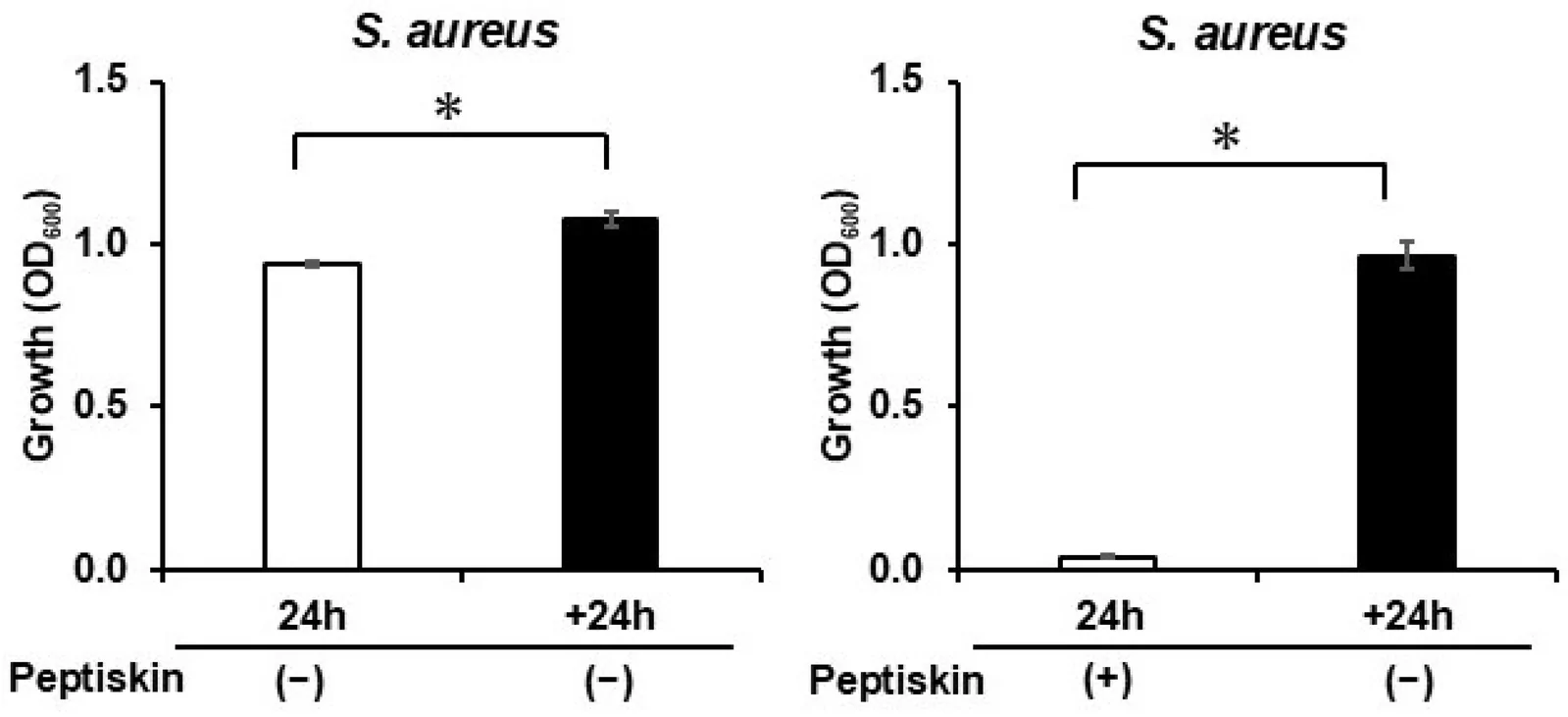
Bacteriostatic effect of Peptiskin on *S. aureus*. *S. aureus* was cultured for 24 h in the absence ((**left panel**), −) and presence ((**right panel**), +) of Peptiskin (0.08%), and turbidity was measured (24 h, □). Subsequently, the bacterial cells were collected, washed with PBS to remove Peptiskin, and resuspended in fresh Peptiskin-free medium. The cells were then cultured at 37 °C for 24 h (+24 h, ■). Turbidity was measured after re-culturing to assess the reversibility of growth inhibition by the Peptiskin treatment. Data are shown as mean ± SD (*n* = 4). * *p* < 0.05.

When *S. aureus* was cultured for 24 h in the presence of Peptiskin, significant growth inhibition was observed compared with that in its absence (Figure 7). In contrast, after removing Peptiskin and replacing it with fresh Peptiskin-free medium, *S. aureus* growth recovered to approximately 80–90% of that in the Peptiskin-free group after 24 h. These results indicate that Peptiskin caused substantial growth inhibition in *S. aureus*, while a fraction of the exposed population remained capable of subsequent proliferation after Peptiskin removal.

### 3.8. Effects of Peptiskin on Intracellular Gene Expression

**Figure 8:**
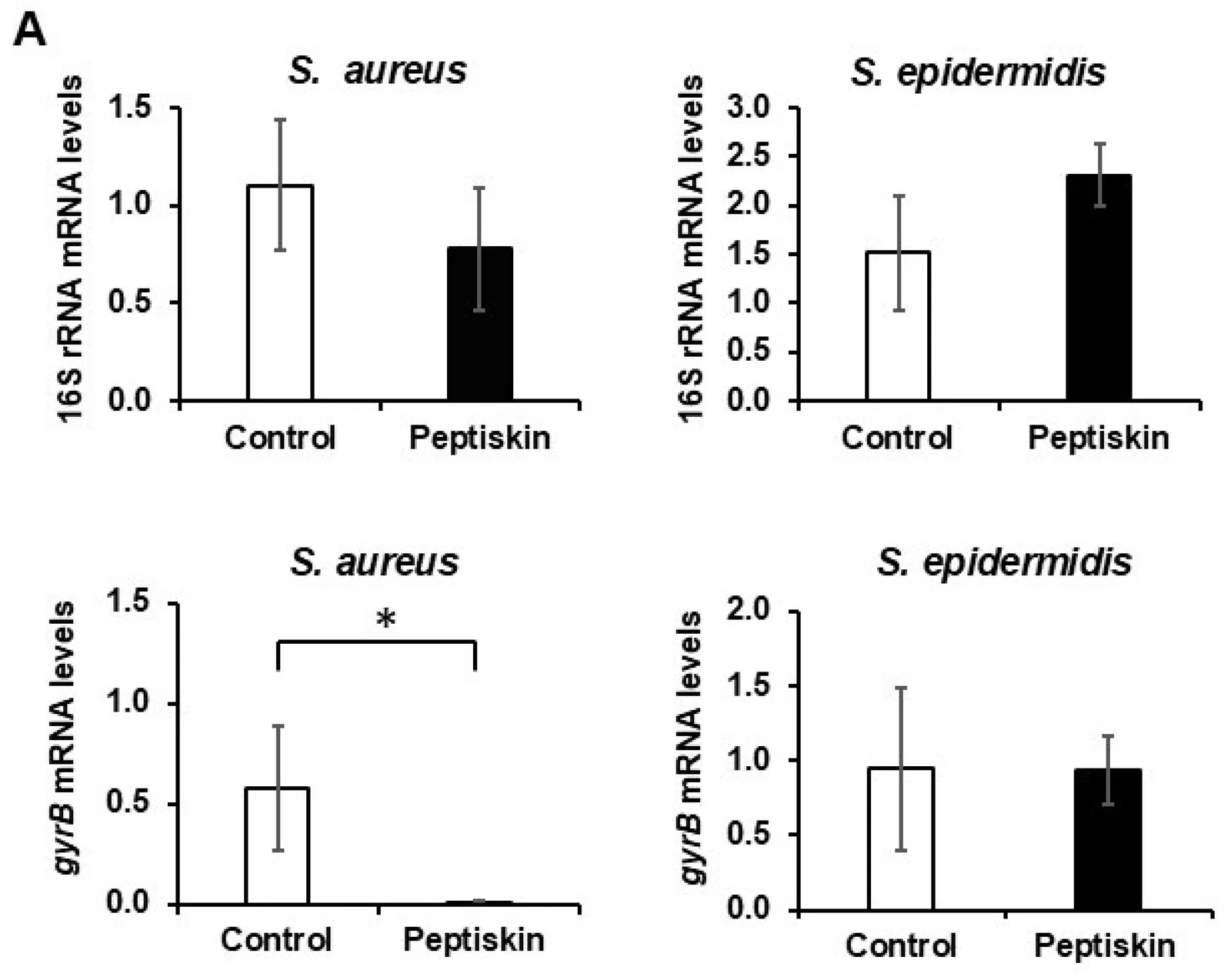

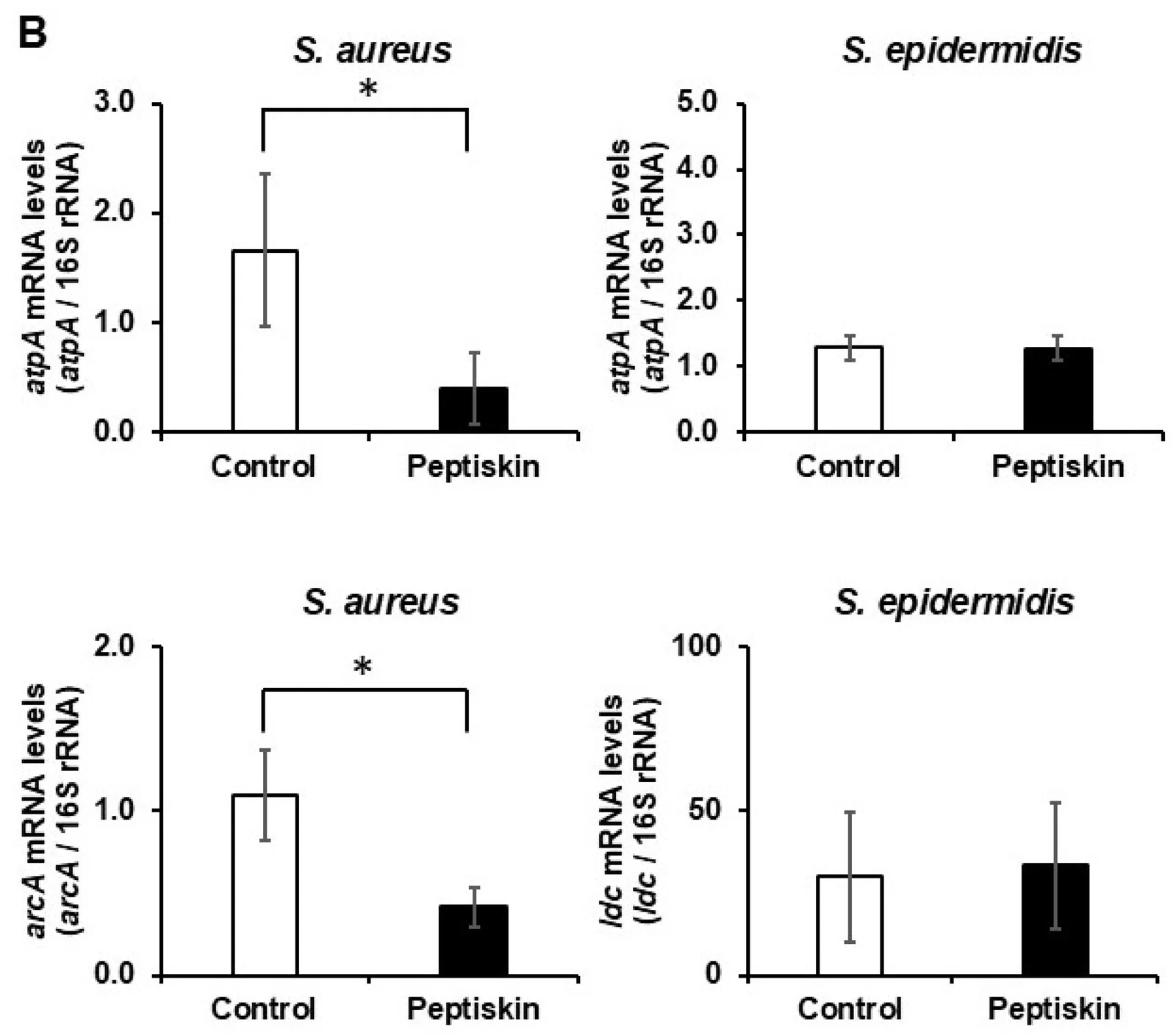
Effect of Peptiskin on gene expression in *S. aureus* and *S. epidermidis*. Total RNA was extracted from *S. aureus* and *S. epidermidis* cultured for 24 h without Peptiskin (□) or with 0.08% Peptiskin (■), and gene expression was analyzed using qRT-PCR. (**A**) The expression levels of 16S rRNA and *gyrB* were compared to identify the internal reference gene. (**B**) Using 16S rRNA as the internal standard, the mRNA expression levels of ATP synthase-related genes (*atpA*, both species), arginine metabolism-related genes (*arcA*, *S. aureus*), and lysine metabolism-related genes (*ldc*, *S. epidermidis*) were analyzed. Data are shown as mean ± SD (*n* = 3–4). * *p* < 0.05.

Total RNA was extracted after 24 h of culture. In *S. aureus*, RNA recovery was significantly lower in the presence of Peptiskin, whereas a significant increase was observed in *S. epidermidis* (Table 2). The total RNA concentrations obtained from untreated and 0.08% Peptiskin-treated *S. aureus* were 5431 ± 1140 ng/µL and 28 ± 9 ng/µL, respectively, whereas those from untreated and 0.08% Peptiskin-treated *S. epidermidis* were 1231 ± 650 ng/µL and 2359 ± 751 ng/µL, respectively (Table 2). Examination of the candidate reference genes by qRT-PCR showed that, although variations in 16S rRNA expression were observed, no statistically significant change was detected in the presence of Peptiskin (Figure 8). In contrast, *gyrB* expression in *S. aureus* was significantly reduced in the presence of Peptiskin. Based on these results, *gyrB* was not considered suitable as a reference gene under the experimental conditions used in this study, whereas 16S rRNA was selected as the reference gene for subsequent analyses. Analysis using 16S rRNA as the reference showed that, in *S. aureus*, the expression of ATP synthase- and arginine metabolism-related genes was significantly reduced in the presence of Peptiskin. These changes were observed in the Peptiskin-exposed *S. aureus* population and may reflect changes in the physiological state associated with exposure to Peptiskin. In contrast, no significant changes were observed in the expression of ATP synthase or lysine metabolism-related genes in *S. epidermidis*.

## 4. Discussion

Through a multifaceted experimental analysis, this study revealed several factors that may contribute to the seemingly contradictory biological responses to Peptiskin, a mixture of short-chain cationic oligopeptides containing arginine and lysine. Peptiskin selectively inhibits *S. aureus* while maintaining and promoting the growth of *S. epidermidis* under identical conditions. These contrasting species-specific responses cannot be fully explained by Peptiskin-induced pH changes or its role as a nutrient source. Instead, the findings suggest that these responses are determined by multiple factors, including differences in eDNA dynamics, cell wall affinity, susceptibility to membrane damage, and metabolic responses.

The proposed mechanisms of action of antimicrobial peptides include pH changes, eDNA trapping, cell wall binding, membrane damage, and inhibition of ATP synthesis [11,18,19,20,21,23,24]. It has also been suggested that eDNA released during cell lysis may serve as a nutrient source for microorganisms [13]. In the present study, we first examined the hypotheses regarding pH changes and nutrient sources (Figure 1 and Figure 2). The pH-adjustment experiment showed that pH can affect the two bacterial species in different directions, with increased growth of *S. aureus* and decreased growth of *S. epidermidis* under the pH-adjusted condition. Thus, a contribution of pH to the observed responses cannot be excluded. However, the pH-adjusted conditions did not reproduce the growth response observed following Peptiskin exposure, indicating that changes in pH alone are insufficient to explain the species-specific effects of Peptiskin. The present experiments were not designed to determine the optimal growth pH for either species; therefore, the optimal pH for *S. epidermidis* cannot be inferred from these data. In addition, the metabolism of Peptiskin and the contribution of bacterial metabolites to the observed changes in culture pH were not directly examined and cannot be completely excluded. Although nutrient-source experiments were performed to investigate the potential use of Peptiskin as a nutrient source, glucose-supplemented positive controls were not included. Therefore, the results suggest that the growth responses observed under the conditions examined cannot be explained solely by the utilization of Peptiskin as a nutrient source (Figure 2).

Analysis of eDNA revealed contrasting responses between the two species. Peptiskin treatment increased the amount of eDNA in the culture supernatant of *S. aureus* by approximately threefold, whereas it decreased that in *S. epidermidis* by approximately half (Figure 3A). Although DNase I treatment promoted the growth of both species (Figure 3C), the amount of eDNA did not correlate with the growth rate. In particular, *S. aureus* exhibited marked growth inhibition despite having significantly higher eDNA levels than *S. epidermidis* (Figure 3A,C). Therefore, the increased eDNA is unlikely to serve as a major nutrient source by itself and may instead be more likely to result from DNA leakage associated with membrane damage and cell disruption caused by Peptiskin.

Interestingly, the addition of DNase I in the presence of Peptiskin partially restored the growth of *S. aureus* and also promoted the growth of *S. epidermidis*. One possible explanation is that eDNA may retain or associate with extracellular molecules or factors that influence bacterial growth. DNase I-mediated degradation of eDNA may alter the availability or distribution of these extracellular factors, which may in turn contribute to bacterial growth even in the presence of Peptiskin. In *S. aureus*, this effect may partially offset the growth inhibition caused by Peptiskin. In *S. epidermidis*, where Peptiskin-induced membrane damage is limited, increased availability of such growth-associated factors may contribute more readily to the maintenance or promotion of growth. However, this mechanism was not directly demonstrated in the present study, and the possibility that DNase I affected bacterial growth through other mechanisms cannot be excluded. Thus, the DNase I experiment provides evidence that eDNA may influence the biological response to Peptiskin rather than establishing a specific molecular mechanism. Furthermore, characterization of eDNA showed that *S. epidermidis*-derived eDNA was predominantly of high molecular weight in the absence of Peptiskin, whereas detectable DNA bands disappeared after Peptiskin treatment, possibly reflecting Peptiskin–eDNA interactions. Conversely, *S. aureus* predominantly contained low-molecular-weight DNA fragments (Figure 3B). These results suggest that Peptiskin interacts differently with eDNA derived from the two species (Figure 3B). An additional experiment in which purified *S. epidermidis* DNA was directly incubated with Peptiskin further supported an interaction between Peptiskin and eDNA, as the addition of Peptiskin resulted in the loss of detectable high-molecular-weight DNA bands by agarose gel electrophoresis. However, this finding does not establish the precise molecular binding mode and cannot distinguish between direct binding, altered electrophoretic mobility, aggregation, or other effects on DNA detection. *S. aureus* produces secreted nucleases that degrade eDNA [30,31]. In *S. aureus*, where membrane damage is substantial, nuclease release associated with cell damage is likely enhanced, resulting in the degradation of eDNA into smaller fragments. In contrast, although the amount of eDNA decreased following Peptiskin treatment in *S. epidermidis*, almost no membrane damage was detected (Figure 3A and Figure 4), and cell viability was maintained. The present data are consistent with the possibility that differences in eDNA processing and degradation contribute to the distinct eDNA profiles of the two species; however, nuclease activity was not directly measured in this study. These findings suggest that the biological significance of eDNA may depend not only on its abundance but also on its molecular weight and mode of interaction with peptides.

Collectively, although the precise molecular mechanism remains unclear, our findings raise the possibility that eDNA may act as a “functional scaffold” that influences the extracellular localization and interactions of Peptiskin. In *S. epidermidis*, which is less susceptible to membrane damage, the interaction of Peptiskin with eDNA may influence its extracellular distribution and availability, potentially contributing to the limited membrane damage and distinct growth response observed in the presence of Peptiskin. The interaction between Peptiskin and eDNA may also alter the local distribution or availability of extracellular molecules associated with eDNA, potentially contributing to the growth of *S. epidermidis*. However, these possibilities remain hypothetical, as the present study did not directly determine the cellular localization of Peptiskin or demonstrate the redistribution of nutrients or other small molecules associated with eDNA. Thus, these extracellular interactions may contribute to the distinct growth responses observed with Peptiskin, although the underlying mechanisms remain to be determined.

Consistent with this interpretation, *S. aureus* exhibited strong cell wall binding, membrane damage, and antibacterial effects (Figure 4, Figure 5 and Figure 6), together with growth inhibition accompanied by increased LDH release and decreased ATP-related gene expression (Figure 7 and Figure 8). These findings suggest that the strong interaction of Peptiskin with the *S. aureus* cell surface may be associated with loss of membrane integrity, which may contribute to the pronounced growth inhibition and antibacterial activity observed in this species. The decreased expression of ATP synthase-related genes should, however, be interpreted cautiously. Because Peptiskin strongly inhibited the growth of *S. aureus*, the RNA recovered after Peptiskin exposure represented the surviving or recoverable bacterial population rather than a population undergoing normal growth. Under the conditions examined, *S. aureus* did not undergo substantial net growth in the presence of Peptiskin, and turbidity remained approximately at the initial level, whereas untreated bacteria underwent normal growth. Increased LDH release and recovery of bacterial growth after removal of Peptiskin further suggest that a substantial proportion of the Peptiskin-exposed population was membrane-damaged, while a fraction remained viable and capable of subsequent proliferation. Thus, the lower total RNA recovery from Peptiskin-treated cultures compared with untreated cultures likely reflects both differences in the bacterial populations resulting from differential growth and the effects of Peptiskin exposure on bacterial cells. Although equal amounts of total RNA were used for reverse transcription and qRT-PCR, the marked difference in total RNA recovery between untreated and Peptiskin-treated *S. aureus* reflects the different bacterial populations obtained after culture, as well as the potential effects of Peptiskin on cellular RNA content or RNA recovery. Importantly, lower RNA recovery should not be interpreted simply as a reduction in the initial bacterial amount, because Peptiskin-treated bacteria did not undergo normal proliferation during the culture period. Therefore, the observed decrease in ATP synthase- and arginine metabolism-related gene expression may reflect both the direct effects of Peptiskin on bacterial transcriptional responses and differences in the physiological state of the surviving or recoverable population. Although the direct transcriptional effects of Peptiskin may contribute to the observed gene expression changes, these changes cannot be interpreted solely in terms of direct transcriptional regulation because the Peptiskin-treated population differed substantially in growth and membrane integrity from the untreated population. The total RNA preparations used for qRT-PCR also showed some variation in A260/A280 ratios, but total RNA concentration was adjusted, and equal amounts of total RNA were used for reverse transcription and subsequent qRT-PCR. Thus, the large difference in total RNA recovery between Peptiskin-treated and untreated cultures was not directly reflected in the amount of RNA used in the qRT-PCR reactions. Nevertheless, differences in bacterial growth, viability, and physiological state between the Peptiskin-treated and untreated cultures remain important considerations when interpreting the observed gene-expression changes.

In addition to eDNA dynamics, differences in the molecular composition of bacterial surfaces may contribute to the species-specific effects of Peptiskin. There are limited reports directly comparing the compositions of cell surface molecules, including teichoic acids and membrane proteins, between *S. aureus* and *S. epidermidis*; therefore, specific molecular differences cannot be identified based on the results of the present study. However, differences in the modification status and density of teichoic acids, which are major contributors to the negative surface charge of Gram-positive bacteria [32,33,34], and differences in the repertoire of surface proteins, including *S. aureus*-associated proteins such as protein A [35], may contribute to the differential interaction of Peptiskin with the bacterial cell surface. These intrinsic differences between the two species should be considered when interpreting the cell wall-binding and membrane damage data.

The novelty of this study lies in integrating multiple extracellular and cellular factors to explain species-specific responses to Peptiskin, rather than attributing the observed effects to a single property, such as cell wall affinity or eDNA binding. Of particular importance is the fact that Peptiskin is not a single-sequence peptide but a mixture of short-chain cationic peptides primarily composed of up to seven arginine and lysine residues. This compositional heterogeneity is an important limitation when interpreting the mechanism of action. The present findings describe the biological effects of the Peptiskin preparation as a whole and cannot necessarily be extrapolated to each individual peptide component. Because the preparation contains multiple short-chain peptides with potentially different sequences and molecular properties, the observed cell wall binding, eDNA interaction, membrane effects, and physiological responses may reflect the combined activities of multiple components rather than the activity of a single molecular species. These structural characteristics may allow Peptiskin to interact with both bacterial membranes and eDNA, with the biological consequences of these interactions differing among bacterial species. In *S. aureus*, strong cell surface interactions were associated with membrane damage and growth inhibition. In contrast, in *S. epidermidis*, eDNA interaction was more evident in the present study, whereas membrane damage was relatively limited, potentially contributing to the distinct growth response observed in the presence of Peptiskin. Conventional studies on cationic antimicrobial peptides have generally attributed their antimicrobial activity to membrane disruption by single-sequence peptides or to a single dominant mechanism of action. In contrast, the present study demonstrated marked differences in Peptiskin interactions with the bacterial cell surface and membrane susceptibility between *S. aureus* and *S. epidermidis*, and that its interaction with eDNA differs in multiple aspects, including quantity and molecular weight. These species-specific differences may reflect the coexistence of membrane- and eDNA-mediated extracellular interactions, with their relative contributions potentially differing among bacterial species and contributing to distinct physiological responses. Although this study could not determine whether the primary target of Peptiskin is the membrane, cell wall, eDNA, or another cellular component, the results suggest that multiple interactions may contribute to species-specific responses. Gene expression analysis provides additional evidence that Peptiskin exposure is associated with different physiological responses in the two species. In *S. aureus*, the observed reduction in ATP synthase- and arginine metabolism-related gene expression was accompanied by strong growth inhibition and membrane damage. In contrast, no significant changes were observed in the expression of ATP synthase- or lysine metabolism-related genes in *S. epidermidis*, in which membrane damage was limited and growth was maintained or promoted. These differences suggest that the two bacterial species respond to Peptiskin through distinct physiological states rather than simply differing in the magnitude of a common antibacterial response. However, the analysis was limited to selected candidate genes related to ATP synthase and arginine or lysine metabolism. Transcriptome-wide analysis, such as RNA-seq, was not performed. Therefore, the present results cannot define the broader metabolic pathways affected by Peptiskin or establish a comprehensive molecular mechanism.

In skin diseases, particularly atopic dermatitis and seborrheic dermatitis, the abnormal proliferation of *S. aureus* contributes to disease exacerbation, whereas *S. epidermidis* helps maintain skin homeostasis [4,16,17,36]. The characteristics of Peptiskin demonstrated in this study—selective inhibition of *S. aureus* and maintenance or promotion of *S. epidermidis* growth—suggest that species-selective modulation of bacterial growth may be of interest for microbiome-oriented applications. The skin microbiome has been increasingly recognized as a potential target for therapeutic and cosmetic approaches [37]. Therefore, Peptiskin warrants further investigation as a candidate material for microbiome-oriented applications. However, the present findings are based entirely on an in vitro two-species experimental system and cannot be directly extrapolated to the complex skin microbiome or to clinical or cosmetic efficacy.

This study has several limitations. First, it was conducted using an in vitro culture system and therefore did not reproduce the complexity of the skin environment, including the stratum corneum structure, sebum components, immune responses, and interactions with other bacterial species. Second, although the results suggest the existence and functional involvement of eDNA–Peptiskin interactions, the molecular binding mode and dissociation mechanism remain unclear. In addition, the cellular localization of Peptiskin and the redistribution of nutrients or other extracellular molecules associated with eDNA were not directly determined, and the proposed “functional scaffold” role of eDNA remains hypothetical. Third, Peptiskin is a mixed system consisting of multiple arginine- and lysine-containing short-chain peptides, and the relationship between its structure and function, particularly in comparison with single-sequence peptides, requires further investigation. Fourth, gene expression analysis was limited to selected candidate genes, and transcriptome-wide analyses, such as RNA-seq, will be necessary to comprehensively characterize the physiological and metabolic responses to Peptiskin.

## 5. Conclusions

Overall, this study indicates that the species-specific effects of Peptiskin are not determined by a single factor but rather reflect multiple interacting factors, including cell wall affinity, membrane susceptibility, and eDNA interactions. These findings suggest that Peptiskin-based approaches may have potential for modulating the balance of the skin microbiota rather than simply eliminating target bacteria. Differences in the relative contributions of cell-surface interactions and eDNA binding may underlie the contrasting biological responses, including growth inhibition in *S. aureus* and growth maintenance or promotion in *S. epidermidis*.

## Figures and Tables

**Table 1:** Growth-promoting or inhibitory effects of Peptiskin on *S. aureus* and *S. epidermidis* under diluted medium conditions.

Nutrition	*S. aureus*		*S. epidermidis*	
	Peptiskin (−)	Peptiskin (+)	Peptiskin (−)	Peptiskin (+)
100%	0.837 ± 0.016	0.053 ± 0.002 *	0.624 ± 0.019	0.794 ± 0.016 *
50%	0.310 ± 0.020	0.043 ± 0.001 *	0.223 ± 0.008	0.417 ± 0.015 *
20%	0.123 ± 0.002	0.043 ± 0.005 *	0.126 ± 0.005	0.175 ± 0.005 *

The growth of *S. aureus* and *S. epidermidis* was evaluated by turbidity (OD_600_) in the absence and presence of Peptiskin (0.08%). Data are shown as mean ± SD (*n* = 4). * *p* < 0.05.

**Table 2:** Effect of Peptiskin on RNA recovery of *S. aureus* and *S. epidermidis*.

Strains	Control Group (ng/µL)	Peptiskin-Treated Group (ng/µL)
*S. aureus*	5431 ± 1140	28 ± 9 *
*S. epidermidis*	1231 ± 650	2359 ± 751

RNA recovery was evaluated in *S. aureus* and *S. epidermidis* cultured in the absence or presence of Peptiskin (0.08%). Data are shown as mean ± SD (*n* = 4). * *p* < 0.05.

## Data Availability

The data presented in this study are available from the corresponding author upon reasonable request.

## Abbreviations

The following abbreviations are used in this manuscript:

ATP	Adenosine triphosphate
bp	Base pair(s)
CFU	Colony-forming unit(s)
DNA	Deoxyribonucleic acid
eDNA	Extracellular DNA
ELISA	Enzyme-linked immunosorbent assay
EDTA	Ethylenediaminetetraacetic acid
LDH	Lactate dehydrogenase
NBRC	National Institute of Technology and Evaluation Biotechnology Research Center
OD_600_	Optical density at 600 nm
PBS	Phosphate-buffered saline
PCR	Polymerase chain reaction
qRT-PCR	Quantitative reverse transcription polymerase chain reaction
RNA	Ribonucleic acid
SDS	Sodium dodecyl sulfate
TBE	Tris–borate–EDTA
Tris-HCl	Tris(hydroxymethyl)aminomethane hydrochloride
UV	Ultraviolet
YNB	Yeast Nitrogen Base
